# Atenção primária à saúde em situações de desastres: revisão sistemática

**DOI:** 10.26633/RPSP.2019.76

**Published:** 2019-09-09

**Authors:** Gisele Cristina Manfrini Fernandes, Raiza Santos Treich, Maria Fernanda Baeta Neves Alonso da Costa, Alexandre Barbosa de Oliveira, Silvana Silveira Kempfer, Roberto Ariel Abeldaño

**Affiliations:** 1 Universidade Federal de Santa Catarina (UFSC) Departamento de Enfermagem, Florianópolis (SC) Florianópolis (SC) Brasil Universidade Federal de Santa Catarina (UFSC), Departamento de Enfermagem, Florianópolis (SC), Brasil.; 2 Universidad de la Sierra Sur, Programa de Pós-Graduação Miahuatlán de Porfirio Díaz (OA) Díaz (OA) México. Universidad de la Sierra Sur, Programa de Pós-Graduação, Miahuatlán de Porfirio Díaz (OA), México.

**Keywords:** Desastres, atenção primária à saúde, pessoal de saúde, planejamento em desastres, prevenção e mitigação, Disasters, primary health care, health personnel, disaster planning, prevention and mitigation, Desastres, atención primaria de salud, personal de salud, planificación en desastres, prevención y mitigación

## Abstract

**Objetivo.:**

Identificar na literatura evidências sobre as intervenções relacionadas à preparação e resposta das equipes de atenção primária à saúde (APS) em situações de desastres relacionados a ameaças naturais.

**Métodos.:**

Revisão sistemática da literatura realizada nas bases PubMed, Scopus, Web of Science, Lilacs, Disasters e Google Scholar. Foram selecionados estudos quantitativos (desenho de caso-controle, coorte ou transversal) que relataram intervenções em populações expostas a desastres de origem natural no contexto da APS.

**Resultados.:**

Seis estudos foram analisados, realizados nas Filipinas, Vietnã, Estados Unidos, Chile e Índia. As intervenções de preparação identificadas incluíram a elaboração de protocolos de preparação de enfermeiros e de educação na comunidade; avaliação de risco de vulnerabilidade da comunidade; e identificação dos serviços de APS para a operação de planos de gestão de desastres. As intervenções de resposta descritas foram ações de tratamento a traumas, prevenção de problemas de saúde e participação em cursos de treinamento.

**Conclusões.:**

As evidências identificadas apontaram que as intervenções de preparo são inadequadas e que o desempenho na capacidade de resposta da APS é fraco.

## Abstract (en)

**Objective.:**

To identify evidence from the literature regarding interventions related to preparedness and response of primary health care (PHC) teams in situations of natural disaster.

**Method.:**

Systematic review of the following databases: PubMed, Scopus, Web of Science, Lilacs, Disasters, and Google Scholar. Quantitative case-control, cohort, or cross-sectional studies reporting interventions in the context of PHC on populations exposed to natural disaster were selected.

**Results.:**

Six studies were analyzed, performed in the Philippines, Vietnam, United States, Chile, and India. The preparedness interventions identified in the studies included the design of nursing preparedness and community education protocols; community vulnerability risk assessment; and identification of PHC services for implementation of disaster management plans. The response interventions described referred to trauma care, prevention of health problems, and participation in training.

**Conclusions.:**

The evidence identified in the literature suggest that preparedness interventions are inadequate, and that the PHC response capacity is poor in situations of natural disaster.

## RESUMEN

**Objetivo.:**

Encontrar pruebas bibliográficas sobre intervenciones relacionadas con la preparación y la respuesta de los equipos de atención primaria de salud (APS) en situaciones de desastre relacionadas con amenazas naturales.

**Métodos.:**

Revisión sistemática de la bibliografía realizada en las bases PubMed, Scopus, Web of Science, Lilacs, Disasters y Google Scholar. Se seleccionaron estudios cuantitativos (de diseño transversal, de casos y testigos, o de cohortes) referentes a intervenciones en grupos poblacionales expuestos a desastres naturales en el contexto de la APS.

**Resultados.:**

Se analizaron seis estudios realizados en Chile, Estados Unidos, Filipinas, India y Vietnam. Las intervenciones de preparación encontradas incluyeron la elaboración de protocolos de preparación del personal de enfermería y de educación de la comunidad; la evaluación del riesgo de vulnerabilidad de la comunidad; y la identificación de los servicios de APS para realizar planes de gestión de desastres. Las intervenciones de respuesta descritas se centraron en el tratamiento de traumatismos, la prevención de afecciones de salud y la participación en cursos de capacitación.

**Conclusiones.:**

Las pruebas identificadas señalaron que las intervenciones de preparación son inadecuadas y que el desempeño en lo referente a la capacidad de respuesta de la APS es deficiente en situaciones de desastre.

Desde a década de 1990, a Organização das Nações Unidas (ONU) tem elaborado estratégias visando à redução do risco de desastres e à gestão que apoie a adaptação às mudanças climáticas. Em 2005, foi criado o Marco de Ação de Hyogo (1), que destacava como objetivo geral o aumento da resiliência de nações e comunidades para minimizar perdas frente aos desastres através de cinco metas essenciais a serem alcançadas até o ano de 2015. O Marco de Sendai para Redução de Risco de Desastres 2015-2030 (2), que sucedeu o de Hyogo, propôs reflexões acerca dos riscos atrelados às mudanças climáticas e discussões para intensificar estratégias que reduzam os impactos dos desastres e promovam comunidades resilientes (3). Nesse marco, destacam-se os efeitos das mudanças climáticas, assim como os efeitos da urbanização e da industrialização das sociedades, particularmente frente às poucas mudanças culturais em relação à prevenção de tais eventos, o que intensifica a vulnerabilidade das populações, especialmente nos países menos desenvolvidos. Nesses termos, há tendência de se acentuarem as relações desequilibradas entre o ser humano e o ambiente, com vieses mais econômicos do que sustentáveis (4).

Os marcos de redução de risco atribuem à atenção primária à saúde (APS) um papel socioambiental na preparação e resposta às situações de desastre, o que motivou o desenvolvimento de pesquisas em saúde pública com foco, principalmente, em riscos geofísicos, vulnerabilidade comunitária e sistemas de alerta precoce (3). É amplamente reconhecido que os sistemas públicos de saúde são projetados para lidar com diversas formas de emergências e desastres, e que os profissionais de saúde da APS fazem parte desse sistema. Destarte, destaca-se que são funções essenciais da saúde pública, sistematizadas pela Organização Pan-Americana da Saúde (OPAS), o desenvolvimento de políticas, o planejamento e a realização de ações de prevenção, mitigação, preparação, resposta e reabilitação para reduzir o impacto dos desastres sobre a saúde da população (5). Atualmente, a Agenda de Saúde Sustentável para as Américas (2018-2030) afirma o compromisso de países membros da OPAS com objetivos que se traduzem em desafios emergentes de saúde pública, regionais e nacionais, coincidindo com os Objetivos de Desenvolvimento Sustentável (ODS). Incluem-se aí planos estratégicos e políticas que atendam o acesso equitativo aos serviços de saúde, também em situações de emergências e desastres (6).

Com efeito, o aprimoramento da resiliência em contexto de desastres é um ponto essencial para o desenvolvimento das governanças nacionais, resultando em práticas de gestão local relacionadas às ações de planejamento e execução e organização de planos voltados às fases de prevenção, preparação, resposta, recuperação e readaptação pós-desastres. O fundamental desse processo consiste na avaliação dessas práticas, dos saberes desenvolvidos e dos treinamentos dirigidos aos profissionais da APS (7).

Análises apoiadas na vulnerabilidade de uma população tendem a ser úteis na avaliação e monitoramento do risco de desastres, fortalecendo a preparação para uma resposta efetiva (8). Em geral, pesquisas anteriores enfocaram diversas temáticas – respostas de emergência, contenção e limitação de danos, produção de diretrizes, planos e protocolos, estudos de caso com lições aprendidas, perspectivas sobre a eficácia das respostas e gerenciamento de emergências e desastres (9) – porém sem ênfase na APS. Partindo-se da premissa de que a abrangência e a eficácia das intervenções da APS auxiliam sobremaneira no entendimento de como essas equipes podem intervir melhor na preparação e na resposta a desastres, o objetivo desta revisão foi identificar na literatura evidências sobre as intervenções relacionadas à preparação e resposta das equipes de APS em situações de desastres associados a ameaças naturais.

## MATERIAIS E MÉTODOS

Realizou-se uma revisão sistemática com base na seguinte pergunta: “quais os resultados das intervenções de preparação e resposta de equipes de cuidados primários de saúde em situações de desastres relacionados com ameaças de origem natural?” A revisão sistemática foi realizada de acordo com os itens do *Statement for Reporting Systematic Reviews and Meta-Analyses of Studies* (PRISMA) (10), com protocolo registrado na plataforma PROSPERO (CRD42018079571).

A pergunta de pesquisa foi estruturada da seguinte forma:

- participantes: profissionais de APS;
- intervenção ou exposição: evidências sobre preparação e resposta das equipes da APS;
- comparação ou controle: não houve;
- medidas de resultado: evidência de atuação das equipes de APS em situação de desastre com ameaças de origem natural;
- tipos de estudos: caso-controle, coorte e transversal.

Os estudos incluídos obedeceram aos seguintes critérios: desenho quantitativo, de caso-controle, coorte e transversal; intervenções em populações expostas a desastres ou com intervenções profissionais da APS; publicação em qualquer ano; e estudos em quaisquer idiomas. Foram critérios de exclusão: desenhos que não fossem de coorte, caso-controle, transversal e quantitativo; estudos sem aderência temática; e estudos que não fossem relacionados à APS.

A pesquisa foi realizada nas bases de dados PubMed (https://www.ncbi.nlm.nih.gov/pubmed/), Scopus (https://www.scopus.com), Web of Science (https://login.webofknowledge.com), Lilacs (http://lilacs.bvsalud.org/en/) e Disasters (https://www.emdat.be/). Fez-se a busca da literatura cinzenta no Google Scholar. A estratégia de busca incluiu as seguintes palavras-chave:* disaster, catastrophe, calamity, earthquake, cyclone, hurricane, flood, fire, tsunami, snowstorm, primary health, primary healthcare, primary care*.

As referências dos artigos selecionados para leitura na íntegra foram revisadas para busca de estudos que não tivessem sido localizados no levantamento inicial. Todas as referências foram gerenciadas no *software* EndNote X7.2.1. Os artigos duplicados foram removidos.

A seleção dos artigos iniciou pela leitura dos títulos e resumos, realizada de forma independente pelos revisores 1 (GMF) e 2 (RST). Nos casos de desacordo, a decisão final quanto à inclusão baseou-se no terceiro revisor (MFBNAC). Após a seleção, os textos completos foram lidos. Os dados referentes às características dos estudos foram extraídos pelos revisores 1 e 2, armazenados em planilhas de *Microsoft Office Excel* 2016 e organizados com auxílio de um instrumento construído pelos pesquisadores, que contempla: características do estudo (autor, ano de publicação, país e objetivo, métodos, análise estatística), características dos participantes (amostra de participantes, população) e características dos resultados (intervenções e/ou principais resultados).

Procedeu-se à avaliação da qualidade individual dos estudos selecionados, com base nos critérios da ferramenta *Strengthening the Reporting of Observational Studies in Epidemiology* (STROBE) (11). O risco de viés também foi avaliado: os artigos com mais de 70% de respostas “sim” aos itens completos da lista STROBE (ou seja, resposta “sim” a todos os subitens, quando presentes) foram categorizados como tendo baixo risco de viés; 50% a 70% de respostas “sim”, risco moderado; e menos de 50% de resposta “sim”, alto risco de viés.

## RESULTADOS

**FIGURA 1.**
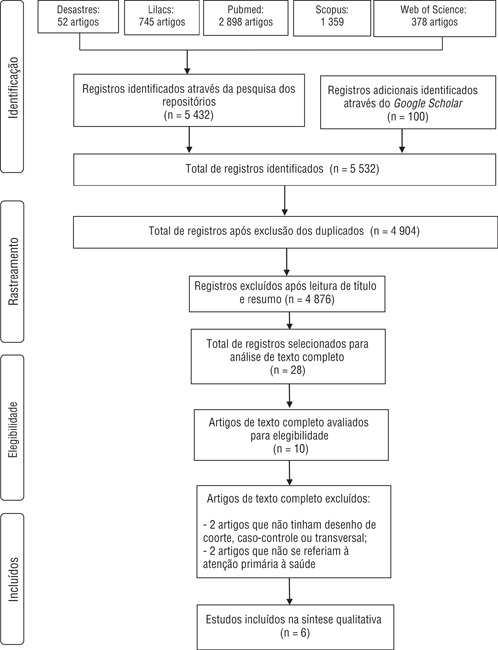
Fluxograma da seleção dos estudos em revisão sistemática sobre capacidade da atenção primária à saúde de atuar em situações de desastres

As buscas identificaram 5 532 artigos. Após remoção de duplicatas e leitura de títulos e resumos, 28 artigos foram selecionados, dos quais 10 foram elegíveis (12-21). Após a leitura integral, foram excluídos dois artigos em função do tipo do estudo (12, 13) e dois artigos que descreviam intervenções sem foco em profissionais de APS (14, 15). Portanto, foram analisados seis estudos que contemplavam os critérios da revisão (figura 1): cinco estudos transversais e um estudo de coorte.

Dos seis artigos selecionados, dois versam sobre preparação a desastres na APS (16, 19), três abordam resposta em desastres (17, 18, 20) e um enfoca mitigação na APS em termos de variáveis de saúde mental da comunidade (21). Quanto à qualidade, quatro artigos tiveram baixo risco de viés (16, 18, 20, 21) e dois artigos tiveram moderado risco de viés (17, 19).

Os estudos foram realizados nos seguintes países: Filipinas (16), Vietnã (17), Estados Unidos (14, 20), Índia (19) e Chile (21). A tabela 1 descreve a síntese qualitativa, listando as principais características dos estudos.

**TABELA 1.** Síntese qualitativa dos dados obtidos por revisão sistemática sobre capacidade da atenção primária à saúde de atuar em situações de desastres

		Amostra^a^					
Referência	País	n	%	Método de coleta de dados	Intervenção	Ameaça	Critério de avaliação
Labrague et al., 2016 (16)	Filipinas	170	85,0	Questionário	Avaliação do nível de preparo dos enfermeiros quanto aos desastres	Ameaças naturais em geral	Preparo
Van Minh et al., 2014 (17)	Vietnã	42	100,0	Questionário (n=22) Entrevista individual (n=10) Entrevista em grupo (n=10)	Concentração na capacidade da APS em responder a desastres com base em modelo da OMS	Tempestades/Inundações	Resposta
Williams et al., 2017 (18)	Estados Unidos	107	23,3	Questionário	Identificação de intervenções para preparação	Ameaças naturais em geral	Resposta
Phalkey et al., 2012 (19)	Índia	29	93,5	Questionário	Reavaliação de planos e ações na contingência e preparação	Inundações	Preparo
Wineman et al., 2007 (20)	Estados Unidos	304	34,0	Questionário	Seleção de alguns itens como indicadores para resposta aos desastres	Tempestades de inverno / frio extremo, tornados e inundações	Resposta
Minoletti et al., 2018 (21)	Chile	549	56,75	Questionários GHQ-12 e SF-36	Relação de intensidade de ações em saúde mental por centros de APS	Terremoto e tsunami	Mitigação

a Amostra: n = número de trabalhadores da atenção primária à saúde convidados a participar da pesquisa; % = proporção de trabalhadores da atenção primária à saúde que completaram as pesquisas.

Foram identificados seis grupos de componentes que interferem na preparação e resposta da APS – serviço assistencial, política/governança, financiamento de saúde, recursos humanos, sistema de informação e pesquisa, produtos médicos e tecnologias – que sintetizam os achados individuais. Os indicadores referiram-se a 1 586 participantes entre os seis estudos selecionados de revisão sistemática. Todavia, grande parte dos indicadores categorizados na tabela 2 foram obtidos de quatro estudos ou menos, por isso a melhor análise que expressa a efetividade ou deficiência nos indicadores se deu individualmente.

O serviço de assistência à comunidade é tratado em quatro dos seis dos estudos. A assistência de saúde à população com serviço de assistência médica e ações preventivas é garantida, de acordo com 39,8% a 50,5% dos profissionais. Os trabalhadores da APS abordaram a forma das atividades médicas e ações preventivas focadas no preparo e resposta a desastres de origem natural, porém mencionaram a possibilidade de algum tipo de limitação (17, 19, 21), como, por exemplo, o encaminhamento a unidades de alta complexidade como um centro cirúrgico (17). Na APS do Chile, de 720 profissionais de saúde estudados, 492 disseram que a população tem cobertura de ações estratégicas de educação e apoio emocional após desastres (21).

As estratégias de governança e políticas foram os indicadores mais estudados: de 1 539 trabalhadores de unidades básicas de saúde, 1 375 (89,3%) declararam ter participado de atividades ligadas à gestão em desastre, como elaboração de planos, protocolos operacionais, protocolo de comunicação e análise de vulnerabilidade (16-18, 20). O conhecimento sobre documentos de prevenção, controle e resposta do governo, assim como sobre protocolos institucionalizados, foi mencionado por 75,4% de 910 participantes dos estudos que trataram do assunto (16, 17, 21). Contudo, três quartos dos profissionais de saúde reivindicavam mais expertise e habilidade em planejamento de desastres e de gerenciamento de plano de emergência (17). Foi mencionada por 705 (79,2%) profissionais a falta de liderança e de compreensão sobre o emprego da APS (20). Além disso, 350 (71,4%) profissionais indicaram a carência de elaboração e implementação das atividades de gestão voltadas às fases de prevenção, preparação e resposta (18, 19). Apenas 108 de 890 profissionais (12,1%) da APS desenvolvem grupos de planejamento de treinamento com participação comunitária (18); 72 de 459 (15,6%) profissionais realizam o processo de identificação de serviços essenciais para a continuidade das operações (18), contribuindo para ações inadequadas de apoio à estrutura e política da APS com intenção de responder a desastres (17).

Os aspectos de financiamento, recursos humanos e alimentação dos sistemas de informação tiveram os menores resultados na análise dos indicadores. A falta de recurso para preparação (17-20) foi mencionada 604 vezes entre 1 400 profissionais (43,1%). Além disso, 178 de 890 profissionais entrevistados (20,0%) indicaram a falta de reembolso dos serviços de emergência (20). O gerenciamento de funções dos profissionais de saúde, ou seja, a re-atribuição de funções (20), aconteceu em 204 de 890 dos casos (22,9%), e a abstinência de profissionais no local de trabalho (19) em situação de desastre foi relatada por 11 de 31 (35,4%) entrevistados. Sistemas de comunicação interrompidos, somados aos sistemas de referência e contrarreferência fora de operação (17), afetaram 11 de 20 centros de saúde que forneciam APS. A presença de serviços de APS que promovem alguma forma de transmissão de informação e educação, tal como *sites* ou memorandos para informar sobre a disponibilidade de vacinas, assim como posto de armazenamento e transporte, foi citada apenas em dois trabalhos por 250 participantes, o que representa 18,5% de atuação para essa atividade (18, 20). A necessidade de melhorar os meios de comunicação, por meio do fornecimento de informações digitalizadas e bancos de dados epidemiológicos regularmente alimentados, foi mencionada por 26 (5,4%) de 479 profissionais de saúde (17, 18).

**TABELA 2.** Indicadores que representam as intervenções das unidades de atenção primária de saúde em situação de desastres conforme revisão sistemática

Indicador	n	%	Freq.
Serviço de assistência à comunidade			
Serviço de assistência médica funcionante (17, 19, 20)	941	50,5	476
Serviço de saúde não interrompido porém com disfunção (19)	31	48,3	15
Encaminhamento para serviço cirúrgico despreparado em caso de emergência (17)	20	65,3	13
Cobertura com estratégia de educação e apoio emocional após o desastre (21)	720	68,3	492
Cobertura populacional e duração de ações comunitárias preventivas (21)	720	39,8	287
Governança			
Participação em atividade relacionada à gestão (plano, protocolo operacional, protocolo de comunicação, análise de vulnerabilidade) (16-18, 20)	1 539	89,3	1 375
Conhecimento sobre documentos de prevenção, controle e resposta do governo, assim como protocolos operacionais preestabelecidos institucionalmente (16, 17, 21)	910	75,4	687
Estrutura e política de apoio a APS para responder a desastres consideradas inadequadas (17)	20	65,0	13
UAPS que desenvolvem grupos de planejamento e participaram de treinamento comunitário (20)	890	12,1	108
Identificação de serviços essenciais para a continuidade das operações (18)	459	15,6	72
Quantia de profissionais que requerem habilidade e expertise em planejamento de desastres, gerenciamento de plano de emergência e encaminhamento de pacientes (17)	20	75,0	15
Falta do desenvolvimento ou implementação de planos, protocolo e/ ou quantidade de treinamento em emergência insuficiente (17, 18)	490	71,4	350
Falha na liderança, coordenação e compreensão do papel da APS pelos planejadores de emergência comunitário (20)	890	79,2	705
Financiamento de saúde			
Falta de recurso para preparação (17-20)	1 400	43,1	604
Falta de reembolso dos serviços de emergência (20)	890	20,0	178
Recursos humanos			
Abstinência^a^ de profissionais (19)	31	35,4	11
Re-atribuição de funções aos profissionais (20)	890	22,9	204
Sistema de informação			
Sistema de comunicação interrompido e sistema de referência e contrarreferência fora de operação (17)	20	55,0	11
Precisa melhorar a comunicação, fornecendo e digitalizando dados da população e epidemiológico (17, 18)	479	5,4	26
Promove informação e educação em saúde (memorando para conhecimento da disponibilidade de parceiros para transporte e armazenamento de vacinas) (18, 20)	1 349	18,5	250
Produtos e tecnologias			
Estabelecimentos têm medicamentos disponíveis (17, 19)	37	83,7	31
Equipamento para primeiros socorros em caso de emergência (20)	890	0,0	0
Outros			
Estrutura física danificada (19)	31	27,5	9
Limitação de vínculo com a comunidade por falta de recurso humano e restrição de tempo (20)	890	34,2	305
Percepção de preparo profissional para situações de desastre			
Totalmente preparado (16, 17)	190	27,3	52
Razoavelmente preparado (16)	170	47,6	81
De certo modo despreparado (16)	170	18,8	32
Totalmente despreparado (16, 17)	190	13,1	25

a Número de profissionais que não compareceram ao atendimentos na atenção primária em saúde em situação de desastres naturais.

Na categoria de produtos e tecnologias, 890 participantes foram questionados sobre a disponibilidade de equipamentos para primeiros socorros em caso de emergência (20). Nenhum relato confirmou a existência desses equipamentos, mas 31 de 37 UAPS afirmaram ter medicamentos disponíveis (17, 19) para atender a população. Um dos artigos analisados mostrou que a dificuldade no desempenho da APS refere-se à limitação de vínculo com a comunidade por insuficiência de recursos humanos e restrição do tempo para as atividades, sendo que 305 profissionais de saúde de 890 (34,2%) indicaram essa ocorrência (20). Outras situações apontadas foram o dano da estrutura física dos estabelecimentos (19) e a percepção do papel e do preparo profissional para situações de desastre (16, 17).

## DISCUSSÃO

Os resultados desta revisão sistemática apontaram que as intervenções existentes de preparo e de resposta a desastres apresentam falhas nas ações de preparação e capacitação da APS. Foram identificados como lacunas: a dificuldade de protocolização da comunicação com a população; e, entre a equipe de profissionais de saúde, a falta de recursos financeiros que atendam às demandas de preparo e de resposta em situação de desastres, o desconhecimento dos profissionais de saúde quanto a estratégias de planejamento e protocolos de emergência existentes e a falta de tempo para desenvolver estratégias junto à população, formular planos e treinamentos. Esses aspectos dificultam a prática voltada ao preparo e resposta da APS diante de ameaças naturais. Entretanto, as próprias evidências apresentam sugestões para transpor essas limitações, principalmente relacionadas ao fortalecimento do vínculo APS-comunidade e da percepção da própria APS sobre seu papel nesse contexto. A evolução da percepção de risco pode se tornar a base para a criação e o desenvolvimento de intervenções bem-sucedidas na APS, facilitando a comunicação com a população.

As falhas podem, também, ser trabalhadas a partir de uma perspectiva coletiva, em conjunto com a comunidade, com atividades de aperfeiçoamento ou criações de *kit* de emergência, incluindo um cartão de informações pessoais de emergência para os membros do agregado familiar; desenho de um mapa de risco na comunidade; desenvolvimento ou melhoria de planos de contingência; e comunicação alternativa para grupos vulneráveis em situações de desastres (22). Tendo em vista as intervenções de preparação, enfatiza-se a relação da APS com os contextos comunitários e a necessidade de gestão de planos contingenciais locais. Há outros estudos na literatura sobre a temática que corroboram tais ações e enfatizam, por exemplo, a importância do conhecimento por parte das famílias de planos de evacuação da comunidade e do estabelecimento de um plano próprio de resposta de emergência familiar (23) que respeite a segurança e otimize a mobilidade em um evento de desastre.

Existem evidências de que a prevalência de estresse pós-traumático, ansiedade e depressão na comunidade permanece elevada 2 anos após a exposição a riscos (24). Essa é uma das razões para o envolvimento dos profissionais em redução de riscos e agravos à saúde que deve ser considerada nas fases de prevenção, preparo e mitigação para melhorar o resultado da assistência (21). O papel das equipes de APS nas comunidades é fundamental na prestação de serviços de saúde essenciais, apontado nos relatórios pós-desastre. Os estudos que validaram intervenções de resposta da APS se relacionavam a tratamentos curativos, medicamentosos ou encaminhamento cirúrgico, ou preventivos dos agravos epidemiológicos consequentes aos impactos dos desastres, considerando que os profissionais saibam a importância de constantes treinamentos e capacitações para o sucesso da operacionalização das intervenções planejadas (16, 20). É preciso capacitar e fortalecer competências profissionais de atuação em cenários de desastre. Essas competências devem contemplar desde a atenção clínica frente às doenças mais comuns a que as pessoas estão suscetíveis até a prática para manobras de emergência, o treinamento para uso adequado dos equipamentos de proteção pessoal e a coordenação dos fluxos de informação durante um desastre (25).

O desconhecimento dos profissionais de saúde sobre os protocolos de emergência existentes está relacionado com a irregularidade no contato com informações que não são rotineiras aos serviços de saúde, a exemplo dos desastres associados a ameaças naturais, possibilitando que o alcance proativo seja reduzido ou negligenciado. Essa situação pode ser contornada a fim de preencher a lacuna de comunicação com a população. Estabelecer estratégias de divulgação prévia ao evento e usar ferramentas que favoreçam a comunicação com a população têm impacto na identificação das necessidades de saúde e no acesso aos serviços de APS em desastres (26, 27).

Um estudo anterior (28) apontou que a resiliência psicológica a eventos climáticos pode diminuir a motivação no sentido de mitigar as mudanças climáticas. Por isso, é importante que as lições aprendidas com eventos de maior magnitude gerem diretrizes e estruturas de coordenação nos planos locais da APS, com integração de setores e ações organizadas pela APS junto à população (20). A integração entre setores oportuniza melhores soluções para os déficits de recursos no preparo, mitigação e resposta para o desastre. Isso garante ao profissional de saúde da APS e à população a operacionalização necessária, facilitando o enfrentamento das barreiras institucionais em torno da gestão e redução de risco de desastres na APS (29, 30).

Voltando à Agenda de Saúde Sustentável para as Américas 2018-2030, há pelo menos três objetivos bem alinhados ao tema que se analisa neste artigo. O primeiro visa à igualdade de acesso aos serviços de saúde, com a devida atenção às necessidades diferenciadas e insatisfeitas de todas as pessoas e às necessidades específicas dos grupos vulneráveis (6). O segundo objetivo trata da inclusão de políticas, planos, normas e processos para a organização do sistema de saúde e mecanismos para seu monitoramento e avaliação (6). O oitavo objetivo, por sua vez, refere-se ao fortalecimento das capacidades de preparação, prevenção, detecção, vigilância e resposta a surtos de doenças e a emergências e desastres que afetam a saúde da população. Apresenta também alguns elementos chaves para seu cumprimento, que podem ser abordados a partir da APS (6): desenvolvimento e fortalecimento da resiliência dos sistemas e serviços de saúde para lidar com emergências e mudanças climáticas; desenvolvimento de capacidades para responder a desastres e emergências causadas por qualquer tipo de ameaça; preparação para emergências e gestão do risco de desastres, incluindo a educação e preparação da população; e fortalecimento do setor da saúde, incluindo recursos humanos e instituições seguras, bem como a preparação de planos nacionais.

Considerando esses aspectos, o presente estudo produz conhecimento sobre a atuação da APS em situações de desastres, revelando indicadores que poderão ser reforçados nas políticas e práticas de saúde em diferentes contextos. Dentre as limitações, reconhece-se que a delimitação do estudo para pesquisas de intervenção e a heterogeneidade da amostra analisada impediram avanços analíticos dos achados da literatura. Pode-se concluir que existe um crescente movimento para mudar a realidade da atuação da APS no contexto dos desastres, mesmo diante das dificuldades quanto ao conhecimento, percepção profissional, desenvolvimento de planos e protocolos e comunicação com a população exposta ou em risco.

### Contribuição dos autores.

Todos os autores (GCMF, RST, MFBNAC, ABO, SSK, RAA) elaboraram o projeto de pesquisa, realizaram a coleta de dados, analisaram e interpretaram os resultados e redigiram o artigo. Todos os autores revisaram e aprovaram a versão final.

### Declaração.

As opiniões expressas no manuscrito são de responsabilidade exclusiva dos autores e não refletem necessariamente a opinião ou política da RPSP/PAJPH ou da Organização Pan-Americana da Saúde (OPAS).
